# Mesoporous Hydroxyapatite/Chitosan Loaded With Recombinant-Human Amelogenin Could Enhance Antibacterial Effect and Promote Periodontal Regeneration

**DOI:** 10.3389/fcimb.2020.00180

**Published:** 2020-04-29

**Authors:** Yue Liao, Huxiao Li, Rong Shu, Huiwen Chen, Liping Zhao, Zhongchen Song, Wei Zhou

**Affiliations:** ^1^Department of Periodontology, Shanghai Ninth People's Hospital, Shanghai Jiao Tong University School of Medicine, Shanghai, China; ^2^Shanghai Key Laboratory of Stomatology, National Clinical Research Center for Oral Diseases, Shanghai Research Institute of Stomatology, Shanghai, China; ^3^State Key Laboratory for Metallic Matrix Composite Materials, School of Materials Science and Engineering, Shanghai Jiao Tong University, Shanghai, China; ^4^Laboratory of Oral Microbiota and Systemic Disease, Shanghai Research Institute of Stomatology, Ninth People's Hospital, Shanghai Jiao Tong University School of Medicine, Shanghai, China

**Keywords:** mesoporous hydroxyapatite, Chitosan, rhAm, periodontal regeneration, antibacterial effect

## Abstract

The recovery of impaired periodontium is still a challenge to the treatment of periodontitis. This study was the first to apply the mesoporous hydroxyapatites/chitosan (mHA/CS) composite scaffold to periodontal regeneration. The aim of our study is to evaluate the biological effects of mesoporous hydroxyapatite/chitosan (mHA/CS) loaded with recombinant human amelogenin (rhAm) on periodontal regeneration. The physicochemical properties of mHA/CS scaffolds were examined by Fourier transform infrared spectroscopy (FTIR), transmission electron microscopy (TEM), and Brunauer–Emmett–Teller (BET) analysis. Then, the biological effects of the mHA/CS loaded with rhAm were evaluated, including antibacterial effect, controlled-release capacity, osteogenic and cementogenic effects *in vitro* and *in vivo*. The antibacterial effect was tested on 1.5 mg/mL CS; 3 mg/mL mHA; 2.25 mg/mL mHA/CS; 4.5 mg/mL mHA/CS and 20 μg/mL rhAm. Tryptic Soy Broth culture medium was used as a baseline control. Osteogenic effect of rhAm (20 μg/mL rhAm), mHA/CS (4.5 mg/mL mHA/CS), and mHA/CS-rhAm (4.5 mg/mL mHA/CS and 20 μg/mL rhAm) on human periodontal ligament cells (hPDLCs) was evaluated in osteogenic media. The hPDLCs treated either with osteogenic media or Dulbecco's modified Eagle's medium (DMEM) alone were used as the baseline control. In the animal model, 4-week-old nude mice (BALB/c) (*n* = 6) implanted with root slices subcutaneously were used to observe the cementogenic effect *in vivo*. The root slices were treated with rhAm (20 μg/mL rhAm), mHA/CS (4.5 mg/mL mHA/CS), and mHA/CS-rhAm (4.5 mg/mL mHA/CS and 20 μg/mL rhAm). The root slices treated with osteogenic medium alone were used as the baseline control. The analyses showed that the mHA/CS particles were 2 μm in diameter and had a uniform pore size. The mesoporous structure was 7 nm in diameter and its surface area was 33.95 m^2^/g. The scaffold exhibited antibacterial effects against *Fusobacterium nucleatum* and *Porphyromonas gingivalis*. The mHA/CS scaffold sustainably released rhAm. The mHA/CS loaded with 20 μg/mL rhAm upregulated ALP activity, the expression levels of osteogenesis-related genes and proteins *in vitro*. Additionally, it promoted the formation of cementum-like tissue *in vivo*. Our findings suggest that mHA/CS loaded with 20 μg/mL rhAm could inhibit the growth of periodontal pathogens and promote the formation of bone and cementum-like tissue.

## Introduction

Periodontitis, as one of the most prevalent oral diseases, which is characterized by the destruction of alveolar bone, which will result in the loosening of teeth and even the loss of teeth (Miranda et al., 2016). Periodontal treatment can eliminate chronic inflammation and infection and stop the progression of disease. However, periodontal defects could hardly be restored to their original form and function (Ivanovski et al., 2014). Therefore, many studies have focused on the use of tissue engineering techniques to achieve the periodontal regeneration, which include three major elements: seed cells, scaffolds, and growth factors (Ogawa et al., 2016).

Periodontal ligament cells have been convinced as the ideal seed cells for periodontal regeneration (Gauthier et al., 2017), which have the capacity of forming cementum-like and periodontal ligament-like tissues (Mrozik et al., 2017). Scaffolds play a vital role in supporting cell adherence and proliferation, maintaining space, and sustainably releasing of growth factors. In addition, the scaffold should be biocompatible and degradable, as it will eventually be replaced by the newly formed tissue (Han et al., 2014; Liu et al., 2016).

A mesoporous structure is a kind of nanostructure with pore sizes ranging from 2 to 50 nm that had attracted great attention in recent years owing to its well-ordered channel system, high porosity, and large surface area (Wu and Chang, 2012; Lee et al., 2016). With the aid of mesoporous structures, scaffolds can load more growth factors and improve bioactivity and cell attachment (Baino et al., 2016).

Hydroxyapatite (HA) is regarded as one of the ideal scaffolds in periodontal tissue engineering. As its similarity to the mineral composition of bones, it has good biocompatibility and bioactive properties (Xiong et al., 2015; Cholas et al., 2016). Thus, mesoporous hydroxyapatites (mHA) scaffold retain the benefits of both hydroxyapatites and mesoporous structures. Experimental studies have shown that nanostructured hydroxyapatites were beneficial to the proliferation and osteogenic differentiation of hPDLCs (Ou et al., 2019), bone marrow mesenchymal stem cells (BMSCs) (Krishnamurithy et al., 2019; Liu et al., 2019; Jose et al., 2020) and adipose-derived stem cells (ADSCs) (Huang et al., 2020). Moreover, nanostructured hydroxyapatites show higher levels of alkaline phosphatase activity than microhydroxyapatites (Domingos et al., 2017). However, compared with the traditional nanohydroxyapatites, few studies have focused on the mesoporous hydroxyapatite.

Despite their benefits, mesoporous hydroxyapatites still have limitations in terms of their mechanical properties, degradation rates and drug release properties (Xiao et al., 2016; Jang et al., 2017). In addition, natural high-polymer materials, such as chitosan (CS), possess high elasticity, and porosity (Rodríguez-Vázquez et al., 2015). Additionally, chitosan has broad-spectrum antimicrobial and anti-inflammatory properties. It has exhibited effective antibacterial activity against several oral pathogens and inhibited the growth of periodontal pathogens (Hu et al., 2019; Zupančič et al., 2019). However, chitosan has inevitable disadvantages, such as the high degradation rate and low mechanical strength (Rodríguez-Vázquez et al., 2015).

When hydroxyapatite and chitosan are combined together as a composite scaffold, it could retain the benefits of good biocompatibility and mechanical strength. The degradation rate is also adjusted. Organic-inorganic nanocomposites have been shown to be beneficial in many fields, such as tissue engineering and drug delivery (Ali and Ahmed, 2017), and a nano hydroxyapatite/chitosan composite scaffold has been used to achieve favorable outcomes for bone regeneration (Deepthi et al., 2016). A positive effect was observed when it was used for periodontal regeneration (Qasim et al., 2015). However, few studies have focused on the biological effects of mHA/CS when used in the field of periodontal regeneration.

Growth factors are also crucial elements in tissue engineering. Enamel matrix proteins (EMPs) have been widely used in periodontal regeneration and have demonstrated the capacity for promoting wound healing, restricting epithelial downgrowth, and supporting the regeneration of a complete periodontal attachment apparatus (Bosshardt et al., 2015; Talebi Ardakani et al., 2019). Amelogenin is the main composition of enamel matrix proteins (Wyganowska-Swiatkowska et al., 2015). The recombinant human amelogenin (rhAm) used in our study was purified from prokaryotic expression system. As periodontitis is a chronic inflammatory disease caused by bacteria plaque, the destructive periodontium is usually under an inflammatory and hypoxia microenvironment, which is unfavorable for the healing of periodontium. So, one of the challenges is to recover the periodontal tissue under the inflammatory microenvironment, and even reverse the inhibitory effect of the inflammatory and hypoxia (Song et al., 2017). The rhAm at a concentration of 20 μg/mL can promote the proliferation of hPDLCs and enhance ALP activity in an inflammatory microenvironment, which indicates that rhAm could improve the osteogenic effect of hPDLCs and compensate for the bone resorption caused by *P. gingivalis* LPS (Dong et al., 2016). The mHA/CS could be an appropriate carrier for rhAm. Firstly, the large specific surface area of mHA could improve loading capacity of rhAm. Secondly, the positive charges from chitosan will be attracted to the negatively charged surface of mHA, which could form a coating layer (Zhang et al., 2016; Feiz and Meshkini, 2019). So, it is suggested that rhAm could be released sustainably accompanied by lower degradation rate of CS with the help of electrostatic interaction between mHA and CS.

It is the first time to apply the mesoporous hydroxyapatite/chitosan loaded with rhAm (mHA/CS-rhAm) to periodontal regeneration. This study was aimed to analyze the physicochemical properties, sustained-release effect and antibacterial effect of mHA/CS against periodontal pathogens. Then the osteogenesis effects of mHA/CS/rhAm was examined on hPDLCs *in vitro*, and cementogenic effects was observed on root slices *in vivo* (Supplementary Figure 1). So, our study is to provide an experimental basis for further investigations in the field of clinical periodontal regeneration.

## Materials and Methods

### Fabrication of mHA and mHA/CS

Briefly, a Cetyltrimethyl Ammonium Bromide (CTAB) solution was mixed with a (NH_4_)_2_HPO_4_ solution. After 3 h of vigorous stirring, CaCl_2_ solution was dripped into the solution (10 drops/ min) at room temperature and stirred for 1 h. The mixed solution was transferred to a reaction kettle to react for 24 h at 100°C. The precipitates were washed with ethyl alcohol for four times, and then dried up in a vacuum-drying oven for 8 h at 40°C. The precipitates were ground into powder. Finally, the mesoporous hydroxyapatites could be obtained after calcination in a muffle furnace for 5 h at 600°C. The Ca/P molar ratio is 5/3.

Mesoporous hydroxyapatite/chitosan scaffolds were synthesized by a hydrothermal method. Chitosan (1 g) was dissolved in acetic acid solution (2 wt%, 50 mL), followed by the addition of mesoporous hydroxyapatites (2 g). Chitosan was mixed with mesoporous hydroxyapatites at a mass ratio of 1:2. After 10 h of vigorous stirring, liquid paraffin (50 mL) and Span 80 (5 mL) were added dropwise into the solution at room temperature. Dilute the 25% glutaraldehyde solution (0.25 mL) with acetic acid solution (2 wt%, 50 mL) before incorporating it into the mixed solution. The precipitates were centrifuged and washed with a chloroform and ethyl alcohol solution for four times. Finally, the mesoporous hydroxyapatite/chitosan composites could be obtained after dehydration in a vacuum-drying oven at 40°C for 8 h.

### Characterization of mHA and mHA/CS

The chemical compositions of the scaffolds were analyzed by Fourier transform infrared spectroscopy (Nicolet 6700, Thermo Fisher Scientific, USA). Then, the surface morphology was observed by objective TEM (JEM-2010F; JEOL Ltd, Japan). The specific surface area and pore size were automatically calculated by an automatic analysis instrument (Autosorb IQ, Quantachrome, USA) via the Brunauer–Emmett–Teller (BET) method.

### Bacterial Culture

*Fusobacterium nucleatum* (*F. nucleatum*; ATCC 25586) and *Porphyromonas gingivalis* (*P. gingivalis*; ATCC 33277) were provided by the Shanghai Research Institute of Stomatology and the Shanghai Key Laboratory of Stomatology, Shanghai Ninth People's Hospital (Shanghai, China). *F. nucleatum* and *P. gingivalis* were maintained in Tryptic Soy Broth (TSB; Bacto™, BD, Sparks, MD, USA) and Tryptic Soy Agar (TSA; Bacto™, BD, Sparks, MD, USA), supplemented with 0.5% yeast extract and 0.5% L-cysteine hydrochloride. In addition, 10 μg/mL vitamin K and 5 μg/mL hemin were added to the culture medium of *P. gingivalis*.

### Antibacterial Activity of mHA/CS

TSB-based culture medium was added into a flat-bottomed 24-well plate. *F. nucleatum* and *P. gingivalis* suspensions at final concentrations of 1 × 10^4^ colony-forming units (CFU)/mL and 1 × 10^7^ CFU/mL, respectively, were added to the culture medium in the presence of 1.5 mg/mL CS, 3 mg/mL mHA, 2.25 mg/mL mHA/CS, 4.5 mg/mL mHA/CS or 20 μg/mL rhAm. As CS and mHA were mixed at a mass ratio of 1:2, 4.5 mg/mL mHA/CS was consisted of 1.5 mg/mL CS and 3 mg/mL mHA. TSB-based culture medium was used as a blank control, and the culture medium containing bacterial suspension was used as a negative control. After 48 h of culture, the antibacterial activity was evaluated on the basis of the OD values at a wavelength of 600 nm.

*F. nucleatum* and *P. gingivalis* biofilms were prepared as the description of Zhou et al. (2016), and the samples were also divided into six groups in accordance with the treatment conditions described above. The bacterial cells were stained with the LIVE/DEAD® BacLightTM Bacterial Viability Kit (Molecular Probes Inc., Eugene, OR, USA). After 15 min, the samples were observed from a confocal laser scanning microscope (CLSM, Leica TCS SP2, Germany).

### Isolation and Culture of Human PDLCs

The isolation and culture of hPDLCs were performed on the basis of the method of Song Z. C. et al. (2012). Informed consent was obtained from the patients prior to acquire samples. The Institute Review Board number for the use of human tissue samples is 2018-120-T98, which was proved by Shanghai Ninth People's Hospital. Human PDLCs were obtained from healthy premolars and impacted third molars. The extracted tooth were washed with sterilized PBS for three times. Then the periodontal ligament tissues were scraped off carefully from the root of extracted tooth with surgical blade, and cultured in Dulbecco's modified Eagle's medium (DMEM, Sigma, USA), which is supplemented with 20% fetal bovine serum (FBS). The DMEM was changed every 5–7 days until confluent cells appeared.

### MTT Assay

The hPDLCs were added (1 × 10^5^ cells/well) to 96-well plates. Then, 4.5 mg/mL mHA/CS was added to the culture media of the mHA/CS group. The samples were cultured for 1, 3, and 7 days. Twenty microliter MTT solution (5 mg/mL, Sigma, USA) was added into each well and incubated with hPDLCs for 4 h. Subsequently, solution in each well was replaced by 150 μl DMSO. After 5 min standing, the plate was shaken slightly for about 5 min before detection. The absorbance was measured at a wavelength of 490 nm.

### Measurement of rhAm Release

The samples were divided into three groups. The mHA, CS and mHA/CS suspension liquids were mixed with 20 μg/mL rhAm. The controlled release effect of mHA/CS was measured according to the approach described by Xia et al. with some modifications (Xia et al., 2018). The mHA and mHA/CS composite particles were immersed in phosphate-buffered saline solution (PBS, Sigma, USA) for 24 h in the presence of 20 μg/mL rhAm. After centrifugation, the mHA and mHA/CS particles loaded with rhAm were added to 1 mL PBS. One hundred milliliters of supernatant were extracted at 1, 2, 4, 8 h, 1, 3, and 7 days. The amount of rhAm in the supernatant was evaluated by an ELISA kit.

### Evaluation of the Osteogenic Effect of mHA/CS

The samples were divided into five groups: group D (Dulbecco's modified Eagle's medium); group C (osteogenic differentiation medium only); group rhAm (osteogenic differentiation medium with 20 μg/mL rhAm); group mHA/CS (4.5 mg/mL mHA/CS and osteogenic differentiation medium); group mHA/CS-rhAm (4.5 mg/mL mHA/CS and osteogenic differentiation medium with 20 μg/mL rhAm). Group C was the control group. The osteogenic induction medium (Cyagen, USA) consists of DMEM with 20% fetal bovine serum, β-glycerophosphate disodium salt (10 mmol/L), dexamethasone (0.1 μmol/L) and ascorbic acid (50 mg/L).

The hPDLCs were implanted (1 × 10^5^ cells/well) into 24-well plates and incubated for 24 h. Then, the samples were divided into four groups as previously described and different culture media were added into each well according to their grouping. The samples were incubated for 7 days before the staining. The ALP activity was determined with BCIP-NBT Alkaline Phosphatase Color Development Kit (Beyotime Biotechnology, China) on the basis of the method described by Dumont et al. (2016). After 7 days' treatment, the hPDLCs were fixed with 4% paraformaldehyde for 30 min, then washed with PBS for three times. Subsequently, the solution of BCIP-NBT was prepared as the instructions. One hundred microliters of BCIP-NBT was added dropwise into each well and incubated for 2 h before the observation of the staining.

Cells from different groups were collected at days 3 and 7 after treatment. The osteogenic genes were evaluated by real-time PCR, including RUNX-2, OPN, and DLX-5. The mRNA level of glyceraldehyde-3-phosphate dehydrogenase (GAPDH) acted as an internal control. The ΔΔCt method was used to determine the expression of the target genes. The primers used in the determination are as follows (Table 1).

**Table 1:** The primers sequence of genes used in RT-PCR.

**Target gene**	**Primer sequence**
RUNX2	Forward: 5′-GCGGTGCAAACTTTCTCCAG-3′
	Reverse: 5′-TCACTGCACTGAAGAGGCTG-3′
OPN	Forward: 5′-CCAGCCAAGGACCAACTACA-3′
	Reverse: 5′-AGTGTTTGCTGTAATGCGCC-3′
DLX5	Forward: 5′-GCTCAATCAATTCCCACCTGC-3′
	Reverse: 5′-AGCCCATCTAATAAAGCGTCCC-3′
GAPDH	Forward: 5′-CGGGAAACTGTGGCGTGAT-3′
	Reverse: 5′-GTCGCTGTTGAAGTCAGAGGAG-3′

*RS, root slice; M, ePTFE membrane; MT, mouse tissue; NFT, new fibrous tissue; NFC, newly-formed cementum-like tissue*.

Samples for Western Blotting were also obtained at days 3 and 7. The primary antibodies used are as follows: RUNX-2, OPN, DLX-5, and GAPDH. Subsequently, secondary antibodies were used for the incubation of PVDF membranes. The protein bands were detected by a chemiluminescence detection system (Gel Doc 200, Bio-Rad Laboratories, USA). The intensities of protein bands were analyzed by Image J software to obtain the gray values, which could be analyzed statistically.

### *In vivo* Experiment

All experimental protocols were performed according to the guidelines in EU Directive (2010/63/EU), which was also approved by the ethical committee of the Animal Care and Experimental Committee of the Shanghai Jiao Tong University School of Medicine (Approval no. SH9H-2019-A499-1). Six 4-week-old nude mice (BALB/c) were used for the experiment. They were raised together for 1 week before the experiment to accustomed to the environment.

The preparation of the root slices was according to the method described by Song et al. (2007). The extracted teeth were subject to careful scaling and root planning. Then, the roots were cut into slices longitudinally. After conditioning with 24% EDTA for 2 min, the root slices were washed with PBS repeatedly and sterilized under ultraviolet light for 4 h.

The samples were also divided into four groups: group C; group rhAm; group mHA/CS, and group mHA/CS-rhAm. The root slices were transported in 6-well plates with one root slice per well. Then, hPDLCs were added (2 × 10^6^ cells/well) into the 6-well plates and cocultured with root slices. The culture medium was added, respectively: group C (2 mL osteogenic differentiation medium/well); group rhAm (2 mL osteogenic differentiation medium + 40 μg rhAm/well); group mHA/CS (2 mL osteogenic differentiation medium + 9 mg mHA/CS/well) and group mHA/CS-rhAm (2 mL osteogenic differentiation medium + 9 mg mHA/CS + 40 μg rhAm/well).

After 7 days of coculture, the root slices were wrapped with polytetrafluoroethylene (ePTFE) membranes and implanted into nude mice subcutaneously under anesthesia. At the eighth week after surgery, the root slices were removed along with the surrounding skin and subcutaneous tissue. The specimens were demineralized and dehydrated. The specimens were stained and observed from the microscope.

### Statistical Analysis

The statistical analyses of the data were performed with SAS 9.3 (SAS Institute, Cary, NC, USA). Analysis of variance (ANOVA) and Student's *t*-test were used to evaluate the data in the different groups. The *in vitro* experiments were repeated for more than three times, and the sample size for animal model was based on the study of (Fawzy El-Sayed and Dörfer, 2017). The statistical differences were achieved at *p* < 0.05.

## Results

### Characterization of mHA and mHA/CS

**Figure 1:**
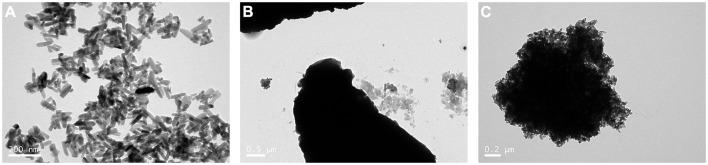
TEM image of mHA, CS, and mHA/CS. **(A)** mHA, observed as short rod-like particles (scale bar 0.2 μm). **(B)** CS, observed as particles with no fixed shape and size (scale bar 0.5 μm). **(C)** mHA/CS, constructed with spherical agglomerates (scale bar 0.2 μm).

The results of the TEM analysis showed that mHA are short rod-like particles that are approximately 20–30 nm in width and 50–100 nm in length (Figure 1A). The particles of chitosan have no fixed shapes (Figure 1B). However, when the mHA particles were combined with chitosan, the composites formed spherical agglomerates with a diameter of 2 μm (Figure 1C).

**Figure 2:**
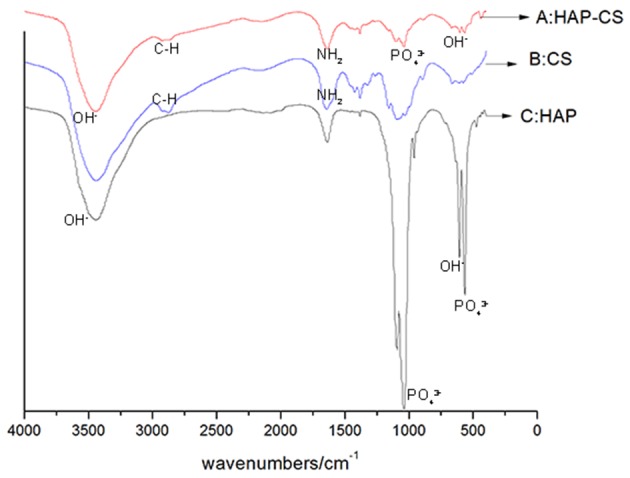
FTIR spectra of mesoporous hydroxyapatite (HAP), chitosan (CS), and mesoporous hydroxyapatite/chitosan (HAP-CS). **(A)** FTIR spectra of HAP-CS, containing characteristic peaks of both HAP and CS. **(B)** FTIR spectra of CS, containing characteristic peaks of C-N and –NH_2_. **(C)** FTIR spectra of HAP, containing characteristic peaks of PO${}_{4}^{3-}$ and OH^−^.

The FTIR spectra of mHA, CS and the synthetic mHA/CS are presented in Figure 2. The spectrum of mHA was characterized by bands at 562 and 1029 cm^−1^, which were assigned to the peak of PO${}_{4}^{3-}$. The peaks at 634 and 3,659 cm^−1^ were the results of the peak of OH^−^. In the spectrum of chitosan, peaks were observed at 2,920 and 2,877 cm^−1^, which contributed to the C-H bond in chitosan. The bands for amide I and amide II were detected at 1,655 and 1,599 cm^−1^. All the characteristic peaks of mHA and CS could be found in the spectrum of the synthesized sample, which means the sample was mHA/CS.

**Figure 3:**
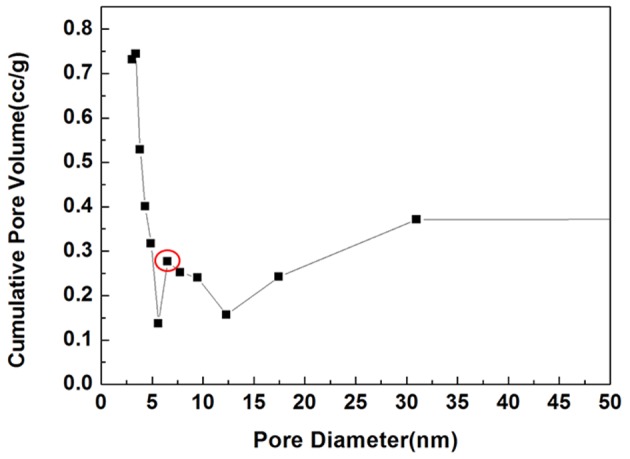
BET image of mHA/CS. Circle, A peak was detected at 7 nm along the horizontal axis, which is the pore size.

As shown in Figure 3, the synthesized sample was mesoporous with an average pore diameter of 7 nm, and a peak was detected along the horizontal axis. The specific surface was 33.95 m^2^/g.

### Antibacterial Effects

**Figure 4:**
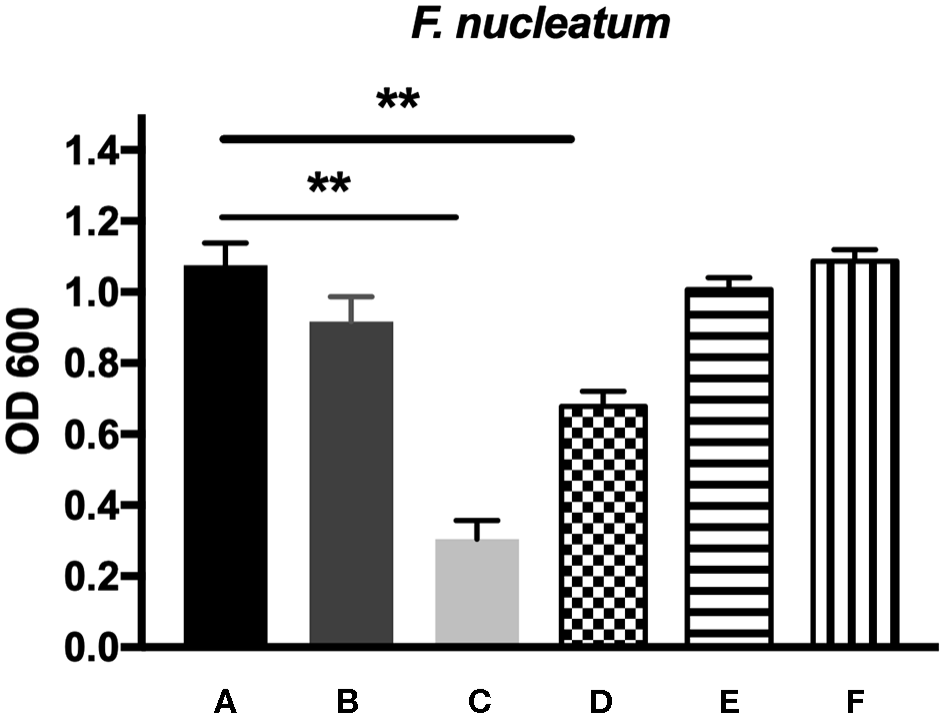
The antibacterial effects of mHA, CS, mHA/CS, and rhAm on 10^4^ CFU/mL *F. nucleatum*. **(A)** Tryptic Soy Broth culture (baseline control), **(B)** 2.25 mg/mL mHA/CS, **(C)** 4.5 mg/mL mHA/CS, **(D)** 1.5 mg/mL CS, **(E)** 3 mg/mL mHA, and **(F)** 20μg/mL rhAm (statistically significant against baseline control, **p < 0.01).

**Figure 5:**
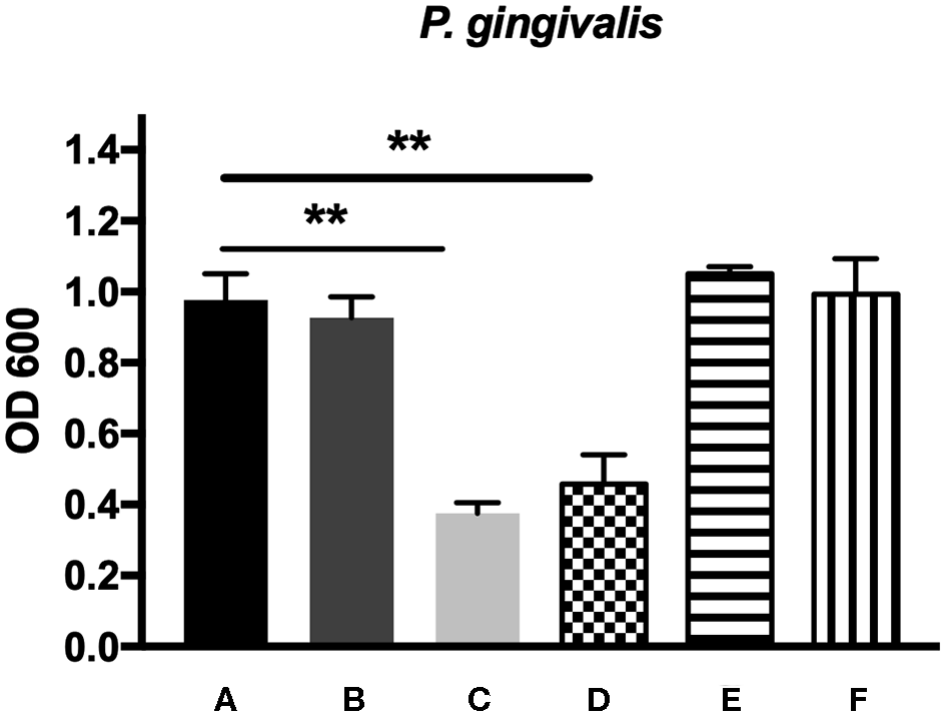
The antibacterial effects of mHA, CS, mHA/CS, and rhAm on 10^7^ CFU/mL *P. gingivalis*. **(A)** Tryptic Soy Broth culture (baseline control), **(B)** 2.25 mg/mL mHA/CS, **(C)** 4.5 mg/mL mHA/CS, **(D)** 1.5 mg/mL CS, **(E)** 3 mg/mL mHA, and **(F)** 20μg/mL rhAm (statistically significant against baseline control, **p < 0.01).

The antibacterial effects of mHA, CS, mHA/CS and rhAm were evaluated on the basis of the OD values (Figures 4, 5). The mHA/CS exhibited dose-dependent inhibition of the growth of *F. nucleatum* and *P. gingivalis*. When mHA/CS was at a concentration of 2.25 mg/mL, the OD values of *F. nucleatum* and *P. gingivalis* showed a declining trend. However, there was no statistically significant difference compared with the control group (*p* > 0.01). When the concentration of mHA/CS reached 4.5 mg/mL, the OD values of *F. nucleatum* and *P. gingivalis* were significantly lower than those of the control group (*p* < 0.01).

The presence of 3 mg/mL of mHA and 20 μg/mL rhAm did not result in antibacterial effects on *F. nucleatum* and *P. gingivalis* (*p* > 0.01). However, 1.5 mg/mL chitosan was shown to decrease the growth of both *F. nucleatum* and *P. gingivalis* in comparison with the control group (*p* < 0.01).

**Figure 6:**
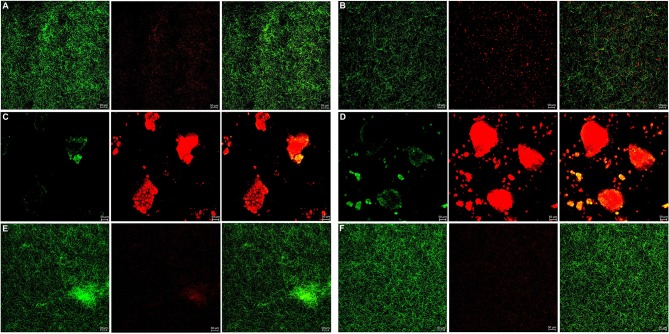
Live/dead fluorescent staining images of *F. nucleatum biofilms*. **(A)** Tryptic Soy Broth culture (baseline control), **(B)** 2.25 mg/mL mHA/CS, **(C)** 4.5 mg/mL mHA/CS, **(D)** 1.5 mg/mL CS, **(E)** 3 mg/mL mHA, and **(F)** 20μg/mL rhAm. Dead cells were stained red, whereas live bacteria were stained green (scale bar 50μm).

**Figure 7:**
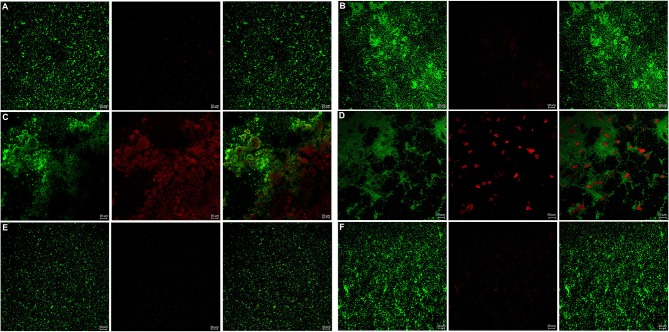
Live/dead fluorescent staining images of *P. gingivalis biofilms*. **(A)** Tryptic Soy Broth culture (baseline control), **(B)** 2.25 mg/mL mHA/CS, **(C)** 4.5 mg/mL mHA/CS, **(D)** 1.5 mg/mL CS, **(E)** 3 mg/mL mHA, and **(F)** 20μg/mL rhAm. Dead cells were stained red, whereas live bacteria were stained green (scale bar 50μm).

The bacterial live/dead fluorescence staining was observed with CLSM to identify the antibacterial effects on *F. nucleatum* and *P. gingivalis* biofilms (Figures 6, 7). The bacteria stained green have intact cell membranes, while the bacteria stained red have impaired cell membranes. It meant that live cells were green and dead cells were red. According to the results, most of the cells were alive in the control group, the 3 mg/mL mHA group and the 20 μg/mL rhAm group. There were several small clusters of dead cells in the 2.25 mg/mL mHA/CS group. While, large amount of dead cells were observed in the 4.5 mg/mL mHA/CS group and the 1.5 mg/mL CS group, which was far more than the live cells in number.

### Examination of Cytotoxicity

**Figure 8:**
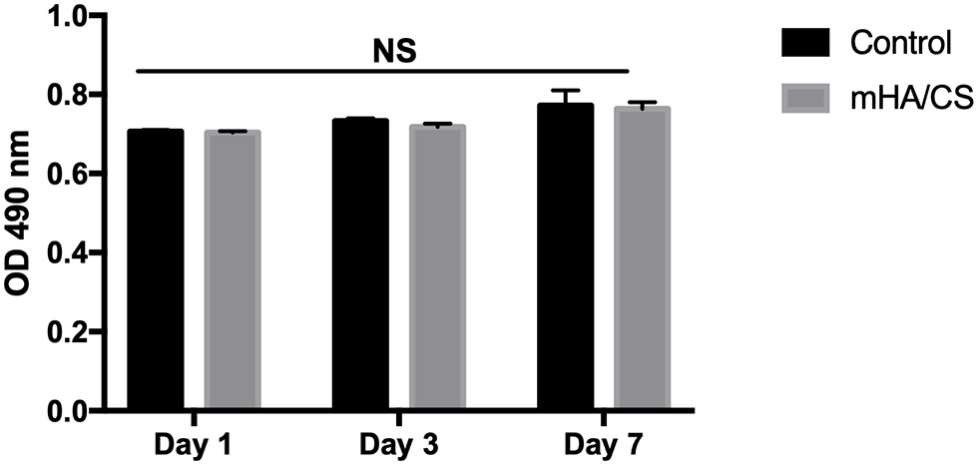
hPDLCs were stimulated by 4.5 mg/mL mHA/CS for 1, 3, and 7 days, and the proliferation was measured by MTT assay. The results showed that 4.5 mg/mL mHA/CS had no cytotoxicity on hPDLCs. NS, no statistical difference.

The cytotoxic effect of mHA/CS on hPDLCs was examined by an MTT assay. On the basis of the results of the assays of antibacterial effects, mHA/CS at a concentration of 4.5 mg/mL was used for the following study. There was no significant difference observed between the mHA/CS and baseline control at days 1, 3, or 7 (Figure 8).

### Controlled-Release Profile

**Figure 9:**
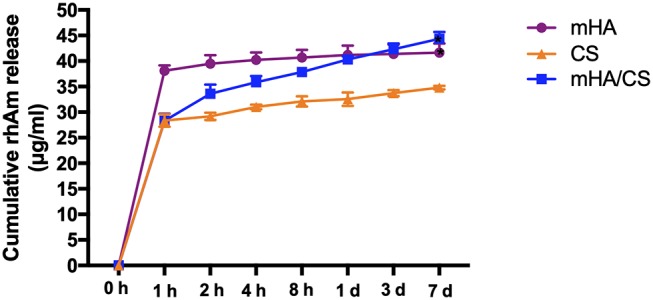
The cumulative release amount of rhAm in group mHA, group CS and group mHA/CS. (Statistically significant against group CS, (*p < 0.05).

The amount of rhAm released from mHA, CS and mHA/CS at selected time points is shown in Figure 9. As the sustained release of rhAm lasted for 7 days, the cumulative release amount of rhAm was calculated according to the results of Elisa test and compared with the release amount at the other time point.

The mHA group exhibited a “release burst” within the initial hour and had a cumulative release percentage of 89%. Subsequently, rhAm was released at a relatively low rate until day 7. However, the early release rate of rhAm in the mHA/CS group was relatively slow. The cumulative amount of rhAm released within the initial hour was 67%, and then the release rate decreased over time until day 7. As chitosan degraded rapidly, 75% of the rhAm was released within the first hour. And then the rhAm was released sustainably as the chitosan was degraded. At day 7, the total amount released by mHA and mHA/CS group was statistically higher than that released by the CS group (*p* < 0.05).

### Alkaline Phosphatase (ALP) Activity

**Figure 10:**
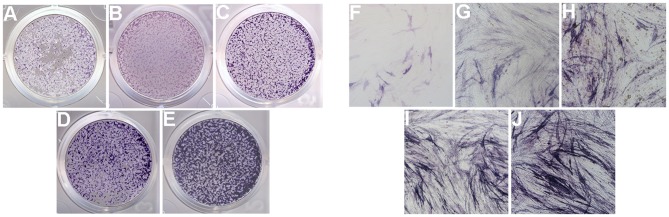
ALP activity of hPDLCs (×100). ALP activity of hPDLCs with the stimulation of DMEM (group D; **A,F**), osteogenic differentiation medium (group C; **B,G**), 4.5 mg/mL mHA/CS (group mHA/CS; **C,H**), 20μg/mL rhAm (group rhAm; **D,I**), and 4.5 mg/mL mHA/CS+20μg/mL rhAm (group mHA/CS-rhAm; **E,J**).

The ALP activity assay was performed to observe the osteogenic differentiation of hPDLCs (Figure 10). The results showed that ALP staining in the group rhAm and group mHA/CS-rhAm was stronger in color than that in the group mHA/CS, C and D. Furthermore, the staining in group mHA/CS was stronger than that in group C and D, and the staining in group mHA/CS-rhAm was stronger than that in group rhAm. The staining in group D was the palest among all the groups. The staining results indicated that hPDLCs in the rhAm and mHA/CS-rhAm groups exhibited stronger osteogenic differentiation capability, which indicated that 4.5 mg/mL mHA/CS loaded with 20 μg/mL rhAm was beneficial to the osteogenic differentiation of hPDLCs *in vitro*.

### Expression Levels of Osteogenesis-Related Genes

**Figure 11:**
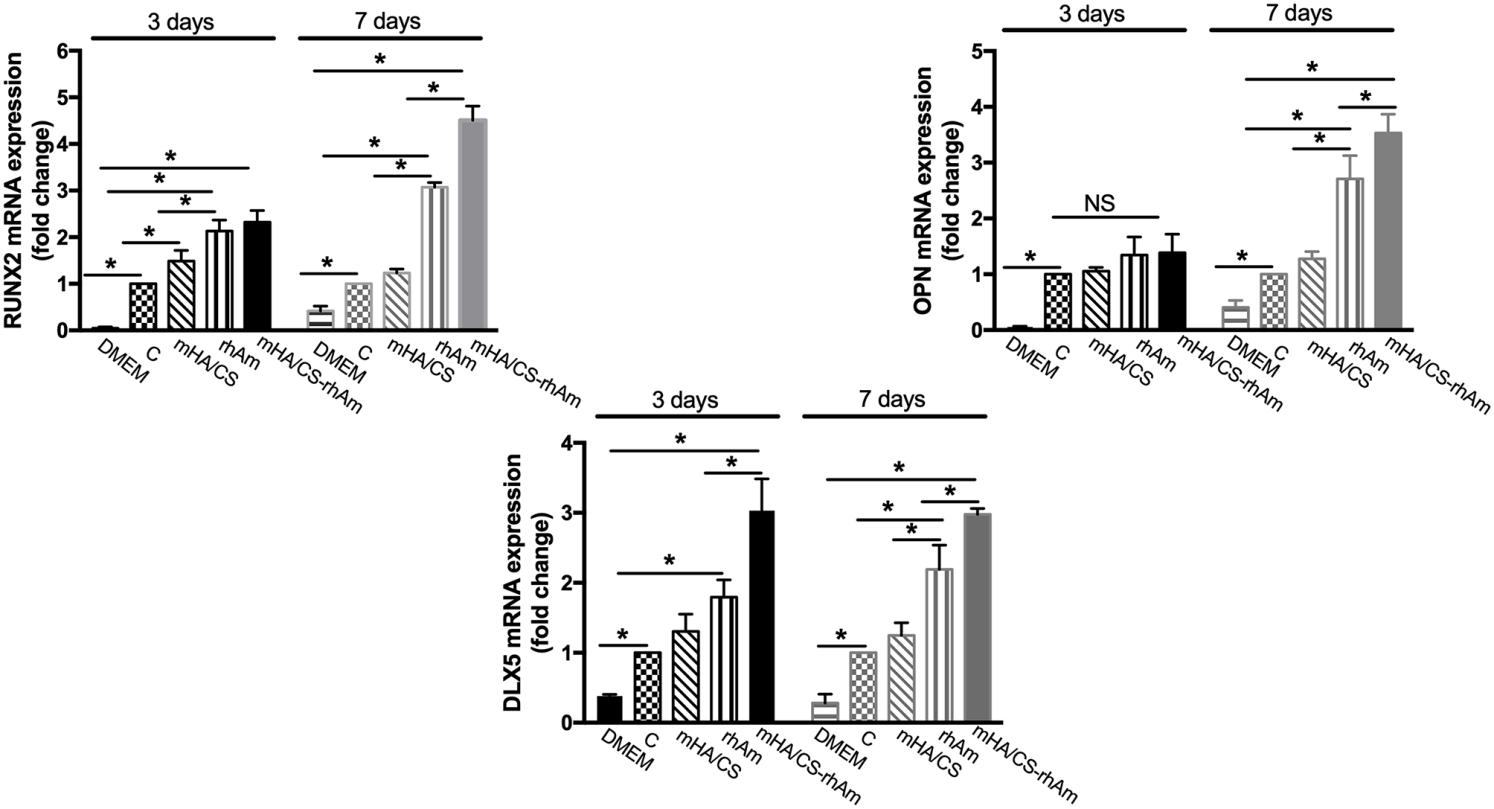
The osteogenesis-related genes RUNX-2, OPN, and DLX-5 were measured by real-time PCR. The RUNX-2 mRNA and DLX-5 mRNA expression level were up-regulated in group mHA/CS-rhAm at both days 3 and 7. The OPN mRNA expression level in mHA/CS-rhAm were up-regulated significantly at day 7 (*p < 0.05).

The expression levels of RUNX-2 and DLX-5 mRNA were significantly upregulated in the mHA/CS-rhAm group when cells were cultured for 3 and 7 days (Figure 11) (*p* < 0.05). In terms of OPN mRNA expression, the mHA/CS-rhAm group exhibited an upward trend at day 3, and the statistically significant differences were observed between the mHA/CS-rhAm group and the C group when cells were cultured for 7 days (Figure 11) (*p* < 0.05). The expression levels of RUNX-2, OPN, and DLX-5 mRNA in group D were significantly lower than those in group C at both days 3 and 7 (*p* < 0.05). Although all osteogenesis-related genes were upregulated in group rhAm, the expressions levels in group mHA/CS-rhAm were the highest among the five groups, demonstrating the osteogenic effects of 4.5 mg/mL mHA/CS loaded with 20 μg/mL rhAm in hPDLCs.

### Expression Levels of Osteogenesis-Related Proteins

**Figure 12:**
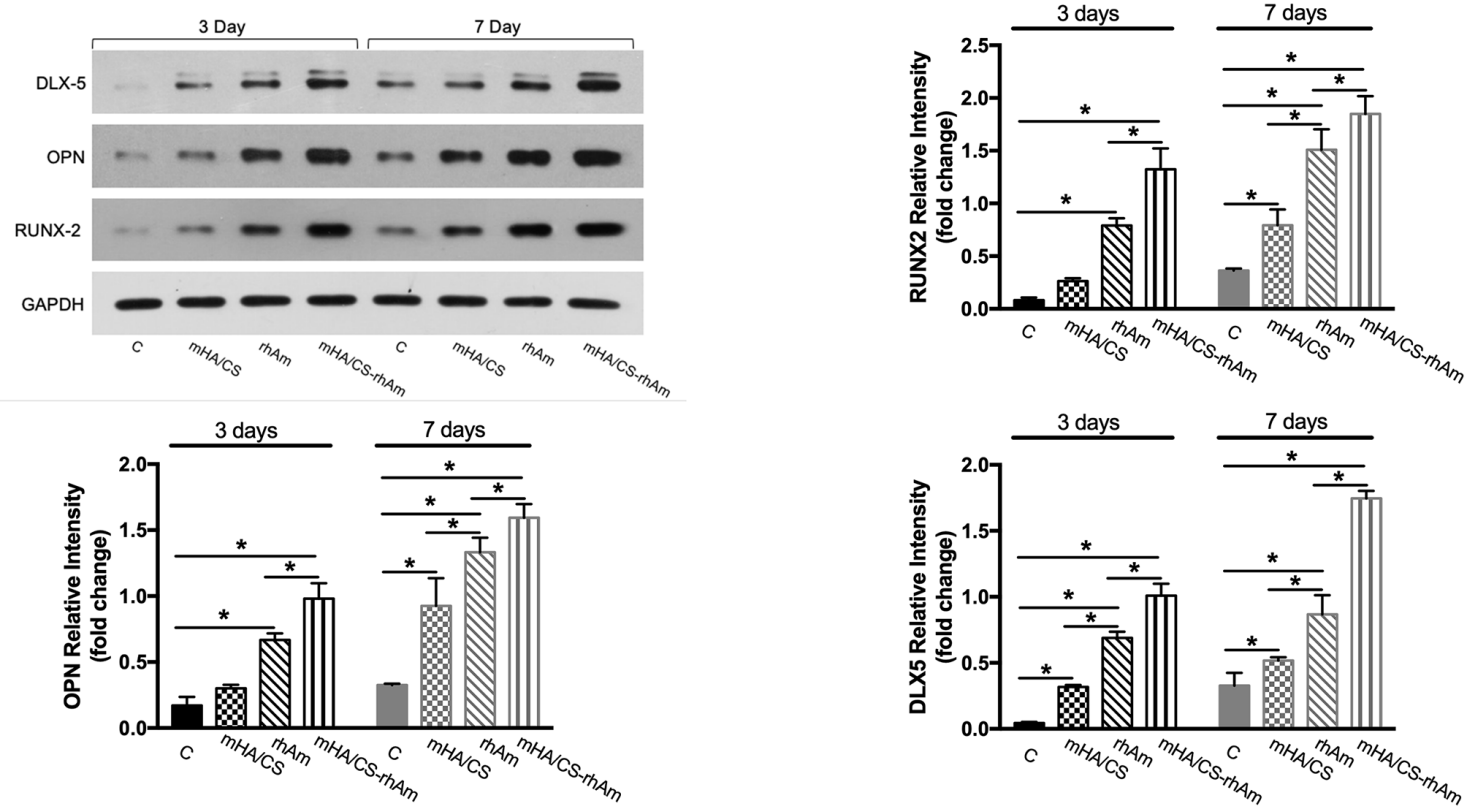
The osteogenesis-related proteins RUNX-2, OPN, and DLX-5 were measured by Western blot at days 3 and 7. The protein expression levels of RUNX-2, OPN, and DLX-5 of hPDLCs in group rhAm and mHA/CS-rhAm were higher than those in the other groups (*p < 0.05).

According the gene expression levels, hPDLCs in group D barely showed osteogenic differentiation potential. So, samples from group D were excluded from Western Blotting. The expressions levels of all osteogenesis-related proteins were significantly upregulated in groups rhAm and mHA/CS-rhAm on both days 3 and 7 (Figure 12) (*p* < 0.05).

Additionally, the protein expression level in group mHA/CS-rhAm was higher than that in the other groups (Figure 12). This result indicated that mHA/CS loaded with 20 μg/mL rhAm could effectively upregulate the expression levels of osteogenesis-related proteins, which was consistent with the gene expression results.

### Histological Observation of Root Slices

**Figure 13:**
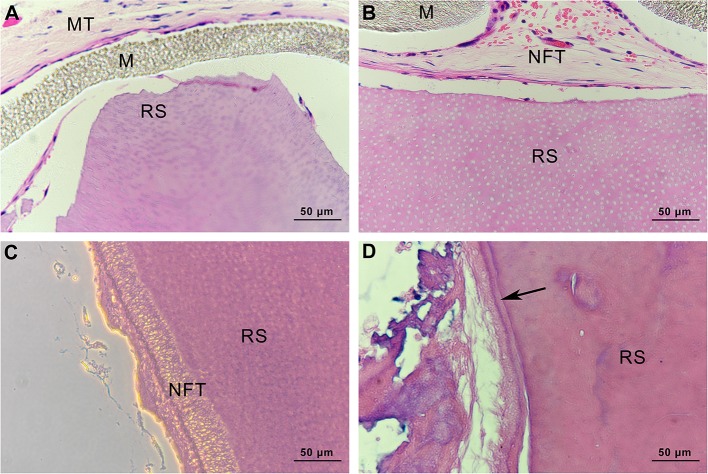
Histological observation of root slices (HE stain, ×400). **(A)** group C. There was no newly-formed soft or hard tissue on the slices; **(B)** group mHA/CS. There was newly-formed fibrous tissue along the inner side of membrane, which had no contact with slices; **(C)** group rhAm. There was newly-formed fibrous tissue along the root surfaces; **(D)** group mHA/CS-rhAm. Newly-formed cementum-like tissue was observed along the root surfaces (arrow) .

In the specimens obtained from group C, there was no soft or hard tissue formation on the root slices. The original cementum had been removed, and the dentine remained. Subcutaneous tissue from mice was observed on the outside of the ePTFE membrane (Figure 13A). In group mHA/CS, new fibrous tissue had formed along the root surfaces and there were large spaces between the newly formed tissue and the root surface. Hard tissue formation could hardly be observed on the specimens. However, no subcutaneous tissue from mice was observed along the inner side of the membrane (Figure 13B). The results suggested that the ePTFE membrane was able to prevent the fibroblasts from entering the membrane and to maintain the space for tissue regeneration. In group rhAm, there was newly formed fibrous tissue containing cells along the root slices. Soft tissue was attached to the root surface with no splits in the space in some areas (Figure 13C). In group mHA/CS-rhAm, there was a thin layer of newly formed cementum-like tissue (NFC) attached to the dentin in most areas. However, there was a split between the fibrous tissue and NFC (Figure 13D).

## Discussion

In recent years, different combinations of scaffolds and growth factors have been applied to the field of periodontal regeneration to repair the periodontium and recover its function (Gümüşderelioglu et al., 2019; Liang et al., 2020). Despite of the achievements in past decades, further studies are still needed to provide a foundation for clinical treatments. The challenges of periodontal regeneration result from the complicated structure of the periodontium and the chronic inflammatory microenvironment. So, our study was aimed to analyze the physicochemical properties, sustained release and antibacterial effects against periodontal pathogens of mHA/CS. Then the osteogenesis effects of mHA/CS/rhAm was examined on hPDLCs *in vitro*, and cementogenic effects was observed on root slices *in vivo*. It is the first time to apply the mesoporous hydroxyapatite/chitosan loaded with rhAm (mHA/CS-rhAm) to periodontal regeneration.

Hydroxyapatite (HA) has been widely used in preclinical and clinical studies of tissue engineering and has components similar to those of natural bone (Gross et al., 2014; Inzana et al., 2014). Compared with the traditional HA, nanohydroxyapatite (nHA) has a larger surface area and better bioactivity and loading capacity. There were sufficient preclinical and clinical findings to support the osteogenic effect of nHA in tissue engineering (Shirmohammadi et al., 2014; Kamboj et al., 2016; Dayashankar et al., 2017; Sadeghi et al., 2017). Chitosan has been used for periodontal treatments in many studies. A 15% Chitosan gel was effective in reducing intrabony defects and improving attachment level (Babrawala et al., 2016). Previous studies have evaluated the osteogenic effects of nHA/CS composite scaffolds (Tu et al., 2017). However, few studies have focused on mHA/CS, especially in the field of periodontal regeneration (Feiz and Meshkini, 2019; Song et al., 2019). On the basis of images of FITR spectra and BET, we synthesized the mHA/CS successfully by the hydrothermal method.

*F. nucleatum* and *P. gingivalis* are the major pathogens that cause periodontitis (Hajishengallis et al., 2012; Yang et al., 2014; Han, 2015; How et al., 2016). Furthermore, coinfection of *F. nucleatum* and *P. gingivalis* could enhance their adherence and invasive capacity in human gingival epithelial cells and inhibit the host innate immune response (Li et al., 2015; Jung et al., 2017). Therefore, we tested the antibacterial activity against both planktonic cultures and biofilms.

From the previous studies, chitosan exhibited antimicrobial activity against not only gram-positive and gram-negative bacteria, but also fungi (Verlee et al., 2017), aim several oral pathogens (Husain et al., 2017; Qasim et al., 2017b). Although Arancibia et al. suggested that chitosan could inhibit the growth of *P. gingivalis* at the concentration of 5 mg/mL, the antibacterial effect of the mHA/CS composite scaffold on periodontal pathogens has not yet been reported. In our study, the OD values indicated that 3 mg/mL mHA and 20 μg/mL rhAm neither inhibited the growth of *F. nucleatum* and *P. gingivalis* planktonic cultures, nor the growth of *F. nucleatum* and *P. gingivalis* biofilms. There were more viable bacteria in these groups which formed dense biofilms. They were stained green from the CLSM images. However, 1.5 mg/mL CS and 4.5 mg/mL mHA/CS had significant antibacterial effects on both planktonic pathogens and biofilms. In the 2.25 mg/mL mHA/CS group, more red staining was observed compared with the control group. The dead bacteria were integrated together and formed into crumb structures instead of dense biofilms. So, the dead bacteria were easily washed away before the staining, which may explain the sparse distributions of staining in 4.5 mg/mL mHA/CS group and 1.5 mg/mL CS group. These results suggested that the mHA/CS scaffold could inhibit the growth of *F. nucleatum* and *P. gingivalis* in both planktonic cultures and biofilms and that the antibacterial effects should be attributed to chitosan. In addition, the MTT assay demonstrated that 4.5 mg/mL mHA/CS has good compatibility with hPDLCs, which could be useful in future studies.

Additionally, chitosan nanocomposites have been developed as prominent materials used for the loading and controlled release of growth factors over time, but few studies have been conducted on mHA/CS (Qasim et al., 2017a; Park et al., 2018). The mHA/CS could be an appropriate carrier for rhAm, which has been proved in our study. As mentioned before, the large specific surface area of mHA could improve the loading capacity of protein. Also, the positive charges from chitosan will be attracted to the negatively charged surface of mHA by electrostatic interaction, which could form a coating layer and slow down the degradation rate of CS. So, it is suggested that rhAm could be released sustainably accompanied by lower degradation rate of CS. In the Figure 9, the cumulative amount of rhAm released by mHA and mHA/CS was constantly higher than CS within 7 days, which confirm that mHA had larger loading capacity of rhAm than CS. Based on the release profile of group CS, 75% of the rhAm was released within the first hour, which was higher than the percentage of group mHA/CS. So, the lowest release amount of group CS implied that CS released less protein than group mHA and group mHA/CS, rather than CS released more slowly.

In our study, the osteogenic effects of mHA/CS loaded with rhAm on hPDLCs were observed *in vitro*. First, ALP activity was observed because of its pivotal role in the regulation of phosphate metabolism and the formation of mineral (Cao et al., 2016). Then, the expression of osteogenesis-related genes and proteins, which represent the different stages of osteogenesis, was detected. RUNX-2 is essential for chondrocyte maturation, matrix production, and mineralization (Liu et al., 2001; Liu and Lee, 2013). As RUNX-2 is the dominant transcription factor in early osteoblast differentiation stage, the statistical differences have already been observed at day 3. The expression levels of RUNX-2 were increased in group H-R at both days 3 and 7. OPN is associated with bone metabolism and remodeling and is an important protein in the middle and late stages of bone formation (Ram et al., 2015; De Fusco et al., 2017). So, there was no statistical significance among four groups at the early stage until day 7. Dlx-5 is regarded as a key regulator of bone formation, and the inhibition of DLX-5 can decrease the expression level of both RUNX-2 and OPN (Samee et al., 2008; Heo et al., 2017). Although, there were no statistical significance between group H and group R at day 3, the expression level of group H-R was upregulated significantly. All the results have proved that mHA/CS loaded with 20 μg/mL rhAm could promote the osteogenic effects *in vitro*.

In the animal model study, only the group containing both mHA/CS and 20 μg/mL rhAm demonstrated the formation of cementum-like tissue that was attached to the dentin with fibrous tissue along the other side. This tissue seems to be similar to that observed in normal periodontium. In the group containing 20 μg/mL rhAm only, there were some newly formed fibrous tissue-containing cells along the root slice. Many factors could influence the outcome of experiment, such as the seed cells, growth factor, root surface condition, and so on. In the research of Song et al., the seed cells were porcine bone marrow-derived stromal cells (BMSCs) and root slices were also derived from pig (Song et al., 2007). While in our study, the seed cells, root slices, and protein were derived from human, so the outcomes of our study would be much closer to the process of clinical periodontal regeneration. According to the HE staining, the old cementum of root slices was removed in our experiment while Song et al. preserved part of the old cementum. Root surface conditioning was essential for the removal smear layer and endotoxin contamination (Karam et al., 2016). However, Song et al. suggested that preservation of healthy root cementum may promote the formation of cementum-like tissue (Song A. et al., 2012). More examinations on the expression levels of genes and proteins related to cementogenic differentiation and animal model studies with extended time span are necessary for the further study.

## Conclusion

In summary, this study demonstrated that the mHA/CS scaffold could inhibit the growth of periodontal pathogens. The cumulative amount of rhAm released by mHA and mHA/CS was statistically higher than CS at day 7 (*p* < 0.05), which confirm that mHA and mHA/CS had larger loading capacity of rhAm than CS. The composite scaffold loaded with rhAm significantly upregulated ALP activity, and the gene and protein expression levels of RUNX-2, OPN, and DLX-5 *in vitro* (*p* < 0.05). Additionally, it successfully induced the cementum-like tissue formation *in vivo*. This study provided a new substitute scaffold for periodontal regeneration that exhibited antibacterial activity and promoted both osteogenic and cementogenic effects.

## Data Availability Statement

The raw data supporting the conclusions of this article will be made available by the authors, without undue reservation, to any qualified researcher.

## Ethics Statement

The studies involving human participants were reviewed and approved by Shanghai Ninth People's Hospital. The Institute Review Board number is 2018-120-T98. The patients/participants provided their written informed consent to participate in this study. The animal study was reviewed and approved by the ethical committee of the Animal Care and Experimental Committee of the Shanghai Jiao Tong University School of Medicine (Approval no. SH9H-2019-A499-1).

## Author Contributions

All authors contributed to the writing of this article. YL, HC, ZS, and WZ conceptualized the study. YL and HL curated the data, conducted a formal analysis, and reviewed and edited the article. ZS and WZ acquired funding. YL worked on the methodology. YL, HL, and LZ administered the project. YL and LZ provided resources. RS, ZS, and WZ supervised the study.

## Conflict of Interest

The authors declare that the research was conducted in the absence of any commercial or financial relationships that could be construed as a potential conflict of interest.

## Supplementary Material

Supplementary Figure 1 The graphical abstract.
