# Supplementary figures and images for: Mesoporous Hydroxyapatite/Chitosan Loaded With Recombinant-Human Amelogenin Could Enhance Antibacterial Effect and Promote Periodontal Regeneration
Source: Front Cell Infect Microbiol. 2020 Apr 29;10:180. doi: 10.3389/fcimb.2020.00180 (PMC7201038; doi:10.3389/fcimb.2020.00180)

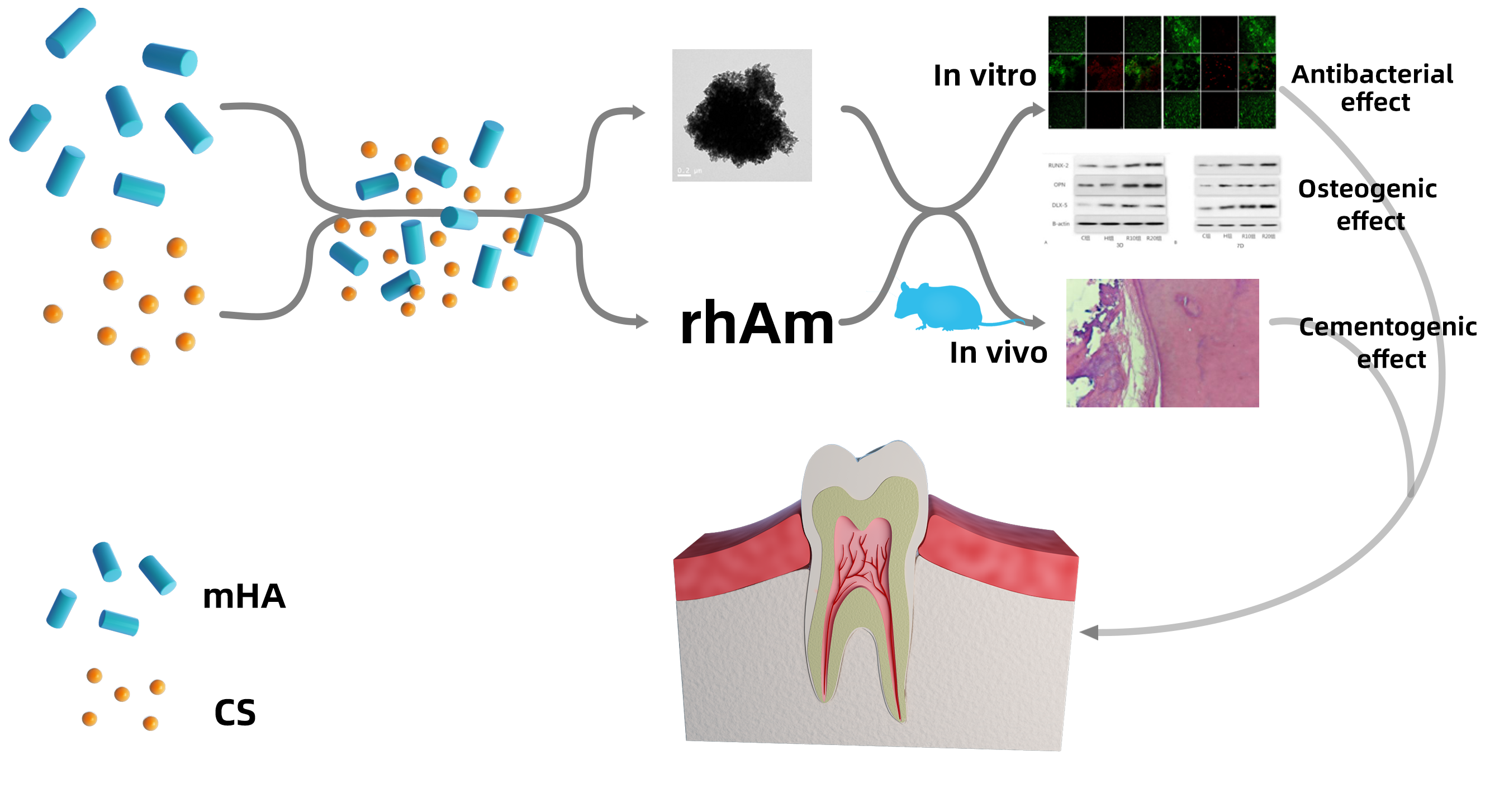

Supplement: Supplementary Figure 1 — The graphical abstract. [file Image_1.TIF]
